# Editorial

**DOI:** 10.1017/qpb.2020.2

**Published:** 2020-08-14

**Authors:** Olivier Hamant

**Affiliations:** Laboratoire de Reproduction et Développement des Plantes, Université de Lyon, ENS de Lyon, UCBL, INRAE, CNRS, Lyon, France

## Abstract

Like many scientific communities, plant science has moved to a new era with the rise of quantitative approaches. This is not merely about high-resolution quantification methods or the generation of massive datasets through omics; the quantitative revolution is much deeper because it unfolds the rich complexity behind plant life.

Like many scientific communities, plant science has moved to a new era with the rise of quantitative approaches. This is not merely about high-resolution quantification methods or the generation of massive datasets through omics; the quantitative revolution is much deeper because it unfolds the rich complexity behind plant life.

As researchers, we have two main missions: to identify good questions and to develop the right strategies to address them. A lot of attention and energy have been spent on the latter one. For instance, we now have robust protocols to perform functional genetics, and even to understand the larger interactions in a cell, an organ or a population. However, by collecting more and more of such data, there is a risk of documenting plant biology, rather than understanding its fundamental rules. Since the turn of century, quantitative plant biology is offering a framework to make sense of that information. Although this new field is often associated with computational modelling or statistical methods, quantitative plant biology is much broader than this. It truly is a revolution that goes beyond a scientific strategy, precisely because it also opens new questions. In other words, the quantitative approach is not only a way to better understand plants, but also has reframed plant science as a whole. This is what the *Quantitative Plant Biology* journal is about.

If asking new questions is the core mission of a researcher, what are good questions? Good questions are often simple. Indeed, answering them can be a highly challenging undertaking, the solution of which can lead to deep insights into nature's big mysteries. For instance, in my area, one of them is ‘how do organs know when to stop growing?’ We have collected millions of data on organ growth, yet, we are still missing a coherent picture. What *Quantitative Plant Biology* hopes to accomplish is to embrace the current revolution in plant science to ask and address such simple questions with complex answers. To do so, we chose three main angles.

First, as new tools are developed to generate massive datasets, be it biochemical, morphological or biophysical, processing the quantified parameters also opens the door to more distant or counterintuitive ideas to test. For instance, how does the variability of cell growth impact shape robustness? Could such fluctuations, through a feedback on growth variability or metabolic modularity, act as an instructive cue to channel morphogenesis? Is this feedback itself regulated, fuelled or dampened by specific pathways? And if so, have these pathways been actively selected during evolution, and how many times? *Quantitative Plant Biology* is opening the Pandora’s box of plant science, and we hope that you, as authors, will shake-up our views on plants.

Second, and beyond research articles, *Quantitative Plant Biology* includes a ‘Theories’ format, specifically designed to explore key questions in plant science in a more prospective way. Computational plant biology has been instrumental in the formalization of testable hypotheses, not only generating credible theories, but also creating new interdisciplinary opportunities for our community with physicists and mathematicians. That said, a good theory does not necessarily need a full computational model, as long as the idea is clearly spelled out, and that the framework for its formalization and testing is shown. Because we, as researchers, ask questions before deploying a strategy, this step is crucial in the process of discovery and it requires a platform to exchange ideas. *Quantitative Plant Biology* will welcome such contributions.

Last, the current quantitative revolution is not restricted to a few elite labs. In fact, massive datasets are collected by citizens all over the world, notably in relation with plant biodiversity or agronomy. Because such data are intrinsically heterogeneous, they also challenge the way plant science works. They are more difficult to handle than data obtained in controlled conditions, but they are likely much more representative of a natural plant environment. Back to research, citizen science challenges our questions, by putting the focus on the societal relevance of our work, as a community. *Quantitative Plant Biology* also aims at being the main platform for this emerging field, notably through the ‘citizen science’ article format.

This ethics translates into the journal itself. *Quantitative Plant Biology* is open access and is supported by not-for-profit institutions (Cambridge University Press and the John Innes Centre). We also aim at enforcing the highest standards in our publications, through the quantitative angle, especially regarding reproducibility.

Why would the plant community need another journal? I hope that these few words have convinced you that *Quantitative Plant Biology* is not just a new journal. It is the forum for a transdisciplinary community that takes plant science for what it is: a systemic, multiscale and open-ended research field with deep societal grounds.

